# Potentiometric Zinc Ion Sensor Based on Honeycomb-Like NiO Nanostructures

**DOI:** 10.3390/s121115424

**Published:** 2012-11-09

**Authors:** Mazhar Ali Abbasi, Zafar Hussain Ibupoto, Mushtaque Hussain, Yaqoob Khan, Azam Khan, Omer Nur, Magnus Willander

**Affiliations:** 1 Physical Electronic and Nanotechnology Division, Department of Science and Technology, Campus Norrköping, Linköping University, SE-60174 Norrköping, Sweden; E-Mails: zafar.hussain.ibupoto@liu.se (Z.H.I.); mushtaque.hussain@liu.se (M.H.); azam.khan@liu.se (A.K.); omer.nour@liu.se (O.N.); magnus.willander@liu.se (M.W.); 2 Nanosciences and Catalysis Division, National Centre for Physics, Quaid-e-Azam University Campus, 45320 Islamabad, Pakistan; E-Mail: yaqoob43@yahoo.com

**Keywords:** honeycomb NiO nanostructures, potentiometric response, ion selective electrode, selectivity, selective ionophore

## Abstract

In this study honeycomb-like NiO nanostructures were grown on nickel foam by a simple hydrothermal growth method. The NiO nanostructures were characterized by field emission electron microscopy (FESEM), high resolution transmission electron microscopy (HRTEM) and X-ray diffraction (XRD) techniques. The characterized NiO nanostructures were uniform, dense and polycrystalline in the crystal phase. In addition to this, the NiO nanostructures were used in the development of a zinc ion sensor electrode by functionalization with the highly selective zinc ion ionophore 12-crown-4. The developed zinc ion sensor electrode has shown a good linear potentiometric response for a wide range of zinc ion concentrations, ranging from 0.001 mM to 100 mM, with sensitivity of 36 mV/decade. The detection limit of the present zinc ion sensor was found to be 0.0005 mM and it also displays a fast response time of less than 10 s. The proposed zinc ion sensor electrode has also shown good reproducibility, repeatability, storage stability and selectivity. The zinc ion sensor based on the functionalized NiO nanostructures was also used as indicator electrode in potentiometric titrations and it has demonstrated an acceptable stoichiometric relationship for the determination of zinc ion in unknown samples. The NiO nanostructures-based zinc ion sensor has potential for analysing zinc ion in various industrial, clinical and other real samples.

## 1. Introduction

Zinc ion is the most abundant heavy metal ion in the human body and the quantity of zinc ion in serum is around 10 μM. Zinc ion is an important constituent of a number of enzymes such as carbonic anhydrase, matrix metalloproteinase [1], and also helps in the maintenance of structural characteristics of gene transcription proteins such as zinc finger proteins, *etc.* [2,3]. In addition to this, high levels of zinc ions are found present in the brain in chelatable form [4], in the pancreas [5], and spermatozoa [6]. The function of chelatable zinc ion is to govern the neuronal transmission in excitatory nerve terminals [4], inhibits apoptosis [7], and leads to neuronal injury under acute conditions [8], epilepsy [9] and transient global ischemia [10]. Zinc ions also stimulate the formation of α-amyloid [11], which causes Alzheimer's disease. Excess of zinc ion can be toxic and pollute the environment as well as decrease the soil microbial activity [12,13]. Zinc ion is also found in food and agricultural wastes [14]. Because of the high quantity of zinc ion in the atmosphere, it can easily be ingested by the human body and may cause pulmonary manifestations, fevers, chills and gastroenteritis. Due to the above facts, it is very important to be able to detect trace quantities of zinc ion and abundant research on this topic is going on in many scientific fields such as medicinal and environmental analysis, *etc.* Many analytical techniques has been used for the determination of zinc ion, including UV-Vis spectroscopy [15], potentiometry [16] and flame atomic absorption spectrometry [17], inductively coupled plasma atomic emission spectrometry (ICPAES) [18] and fluorescence methods [19,20]. These techniques have some limitations due to the completely filled d-orbital of zinc ion, which results in an absence of suitable spectroscopic or magnetic signals. Beside these analytical tools, ion selective electrodes (ISEs) are comparatively simple, cheap and fast. Many zinc ion selective electrodes based on different ionophores have been reported [21–26]. Currently, different selective and sensitive polyvinyl chloride (PVC) membrane-based ISEs for different metal cations have been reported [27–31] and a zinc ion sensor based on functionalised ZnO nanorods has also been published [32]. Moreover, a Schiff's base has also been used for the detection of zinc ion [33].

Recently, the research trend towards nanomaterials is rapidly increasing due to their unique and excellent properties and versatile applications as compared to their bulk devices. The most distinguishing behaviour shown by nanostructures is their dimension-based excitation and emission. The electrical, optical, magnetic, and thermoelectric properties of solid-state functional materials can be controlled by the quantum confinement of electrons through the potential well of nanoscale based structures. Therefore, it is potentially important to grow such nanostructures which are very important for modern science and technology [34–37]. Among the various nanomaterials nickel oxide (NiO) is attractive to researchers due its tremendous properties such as wide band gap (3.6–4.0 eV) [38], magnetic, optical, and catalytic and electrochromic properties [39]. Nickel oxide nanostructures are of great interest for the development of electrochemical energy-storage tools due to their large specific surface area, rapid redox reactions and lowered diffusion path in the solid form. Nickel oxide is also used as magnetic storage material [40], optical active counter-electrode [41], in dye-sensitized solar cells [42], electrochromic films [43] and gas sensors [44,45]. Many methods have been reported for the synthesis of NiO nanostructures such as thermal evaporation [46], RF magnetron sputtering [47], and spray pyrolysis [48]. These growth methods have some limitations such as complex growth processes and the need for high growth temperatures. The size and morphology of the nanostructures is related to the type of technique used for their growth. By changing the growth parameters such as temperature, concentration, growth time, composition of sample solution using different amines, solvents and surfactants the nanostructures of different diameter, different morphologies can be obtained [49].

In the present research work, the hydrothermal growth method was selected for the growth of NiO nanostructures due to its versatility in growing nanostructures of various morphologies, ease, simplicity, environmental friendliness, cheapness and low temperature growth conditions [50]. Besides this, NiO nanostructures grown in the absence of organics at low temperature have also been reported [51]. We have grown honeycomb nanostructures of NiO on nickel foam without the use of any organic compound and the grown NiO nanostructures have been applied for the chemical sensing of zinc ion. This work provides an alternative approach for the further refinement of NiO nanostructures and their industry-based applications as well as use in chemical sensing.

## 2. Materials and Methods

### 2.1. Materials

Nickel sulphate heptahydrate (NiSO_4_·7H_2_O), 25% ammonia (NH_3_), zinc nitrate [Zn(NO_3_)_2_·6H_2_O] ionophore (12-crown-4), sodium tetra phenyl borate (NaTPB), di-n-butyl-phthalate (DBP), tetrahydrofuran (THF) and polyvinyl chloride (PVC) were purchased from Sigma Aldrich Sweden (Stockholm, Sweden). All other chemicals used were of analytical grade.

### 2.2. Fabrication of NiO Honey Comb Nanostructures

The honeycomb-like NiO nanostructures were fabricated on nickel foam substrates using NiSO_4_·7H_2_O and NH_3_ as primary chemical reactants. The nickel foam substrates were sonicated in an ultrasonic bath for 15 minutes using an ethanol solution. Then, the nickel foam substrates were cleaned with deionized water and dried in air. Afterwards the substrates were affixed in a Teflon sample holder and vertically dipped into a mixture of 0.1 M NiSO_4_·7H_2_O and 0.1 M NH_3_ solutions prepared in deionized water. The sample solutions were kept in an oven at 90 °C for 7 hours. The role of NH_3_ in growth process is to act as a complexing reagent for nickel. When the growth time was completed then the substrates were taken out from the oven and a visible greenish colour type film was visible on the nickel foam, then the sample substrates were washed with deionized water and dried in an oven at 80 °C for 3 hours. After drying, some of the as-prepared nickel foam substrates were annealed in air at 500 °C in order to achieve NiO honeycomb nanostructures.

### 2.3. Functionalization of NiO Honey Comb Nanostructures with a Selective Zinc Ion Ionophore (12-Crown-4 Ether)

The functionalization of the honeycomb-like NiO nanostructures with a selective zinc ion ionophore was performed as followed: the ingredients and amounts of each for the preparation of the membrane composition were as follows: 12-crown-4 (40 mg), NaTPB (40 mg), PVC (125 mg), and DBP (100 mg) in tetrahydrofuran (12 mL) [32]. Afterwards the NiO electrodes were dipped into membrane solution for 5 minutes using the direct adsorption method and dried at room temperature for 1 hour then the functionalized electrode were kept in a refrigerator overnight at 4 °C.

### 2.4. Potentiometric Measurements

The potential measurements were carried out for the range of 0.0005 mM to 100 mM of zinc nitrate concentrations prepared in 1 mM phosphate buffer solution of pH 7.3 at room temperature. The functionalised NiO honeycomb-like nanostructures were used as working electrode and silver-silver chloride (Ag/AgCl as reference electrode. A model 744 pH-meter (Metrohm, Switzerland) was used for the potential measurements and a Keithley 2400 (Tektronix, Beaverton, OR, USA) source meter was used for the response time measurements.

## 3. Results and Discussion

### 3.1. Characterisation of NiO Honeycomb Nanostructures

**Figure 1.**
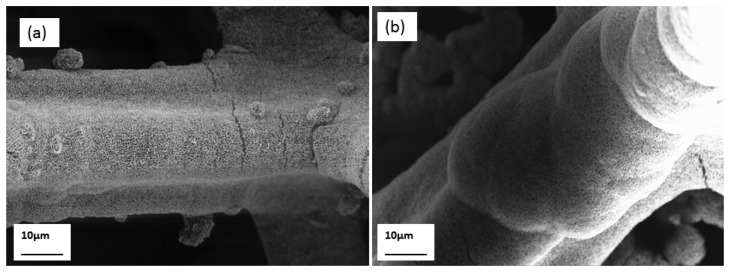

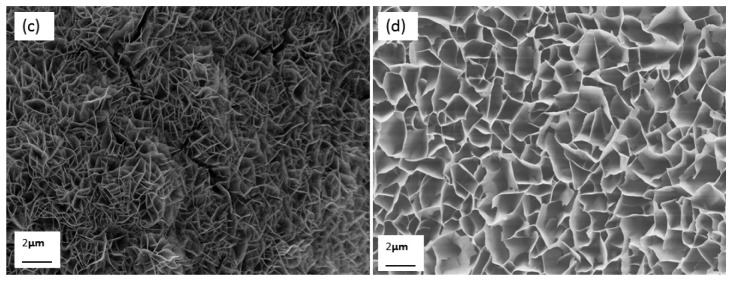
(**a**–**d**) FESEM images of honeycomb-like NiO nanostructures at different magnifications.

The grown honeycomb-like nanostructures were characterised by FESEM, HRTEM, and XRD techniques. In Figure 1(a–d), low magnification FESEM images of the honeycomb-like nanostructures of NiO are shown, which demonstrated the attachment of NiO thin films on the nickel foam substrate. The honeycomb-like NiO nanostructures were clearly seen at high FESEM magnification and from this it can be seen that the grown NiO nanostructures are highly uniform and dense.

**Figure 2.**
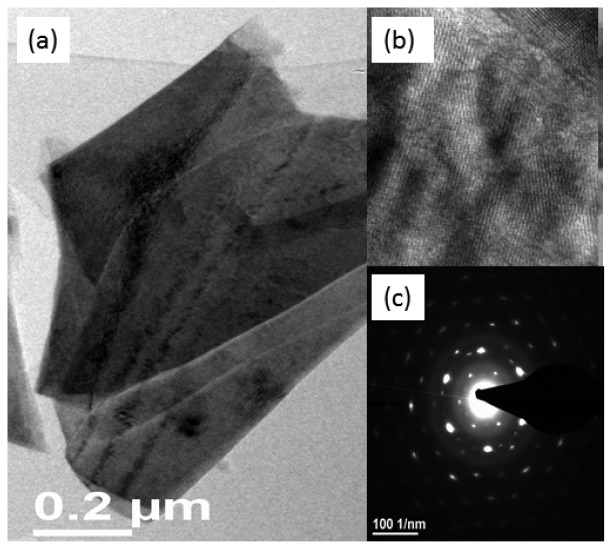
(**a**–**c**) TEM images of NiO nanostructures.

The x-ray diffraction study showed that the nickel oxide honeycomb nanostructures exhibited a face centred cubic phase crystalline structure as shown in Figure 2 and the pattern of XRD is supported by literature data (JCPDS NO. 47-1049).

**Figure 3.**
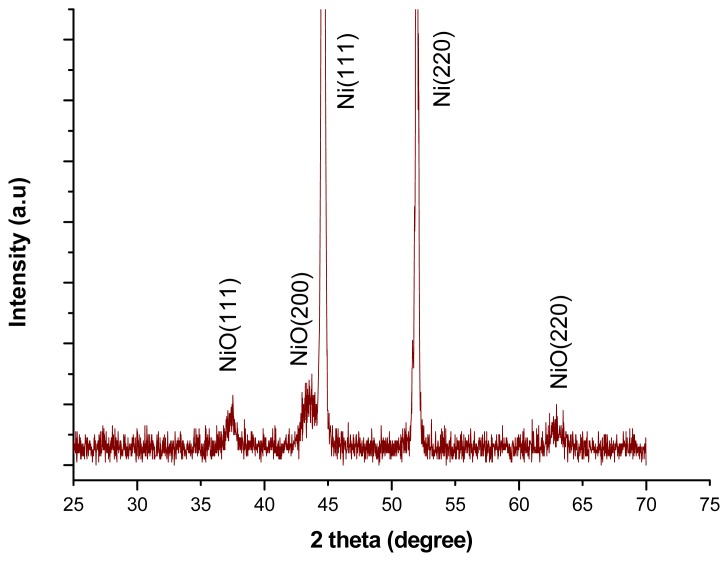
XRD pattern study of the NiO nanostructures.

The transmission electron microscopy study revealed that the NiO honeycomb nanostructures possessed face centred cubic crystallinity with a space lattice of 0.21 nm for the (002) crystal plane of NiO, as shown in Figure 3(a). In the selected area electron diffraction pattern (SAED, Figure 3(b)), it has been observed the results are almost consistent with the XRD patterns; therefore the honeycomb-like nanostructures of NiO are cubic in crystallinity with the presence of rings in the nanostructures described the nanoflakes nature of NiO as shown in Figure 3(c). The crystal size of NiO was calculated by XRD using Scherer's equation for 200 crystal plane and the obtained value for the crystal size is 62 Å.

### 3.2. Potentiometric Response Measurements of the Honeycomb-Like Functionalised NiO Nanostructures

**Figure 4.**
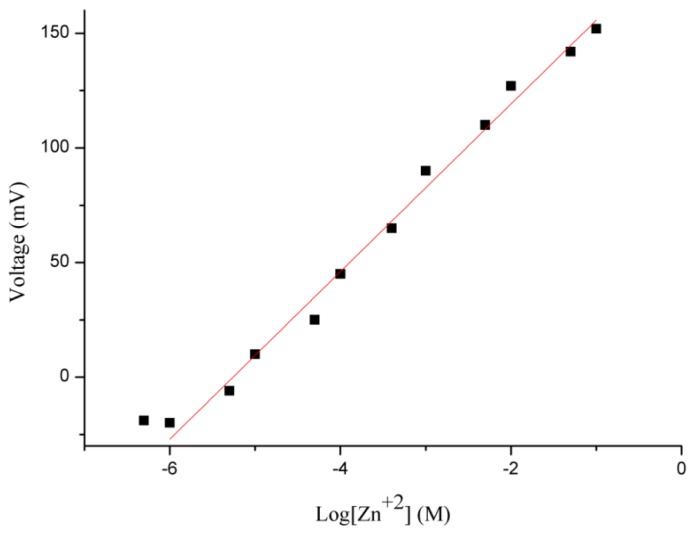
Calibration graph of zinc ion sensor from 0.0005–100 mM zinc nitrate concentrations.

The potentiometric response of the honeycomb-like functionalised NiO-based zinc ion selective electrode was measured for the zinc ions concentration range of 0.0005 to 100 mM. The sensor electrode detected 0.0005 mM concentration of zinc ion, but it was out of the linear range. After the 0.0005 mM concentration of zinc ions, the proposed sensor has shown a highly linear response for 0.001 mM to 100 mM concentrations of zinc ions, as shown in Figure 4.

A sensitivity of 36 mV/decade for the functionalised NiO nanostructures-based zinc ion sensor was observed, with a regression coefficient of r^2^ = 0.99. This is due to the high surface provided by the honeycomb-like nanostructures for the attachment of the selective zinc ion ionophore and due to shortened diffusion path in the solid phase of the NiO nanostructures which might be responsible for the slightly higher slope value than the theoretical value. These two characteristics of the proposed sensor electrode confirmed its potential applicability for analytical purposes.

### 3.3. Working Performance of the Honeycomb-Like Functionalised NiO Nanostructures-Based Zinc Ion Selective Electrode

**Figure 5.**
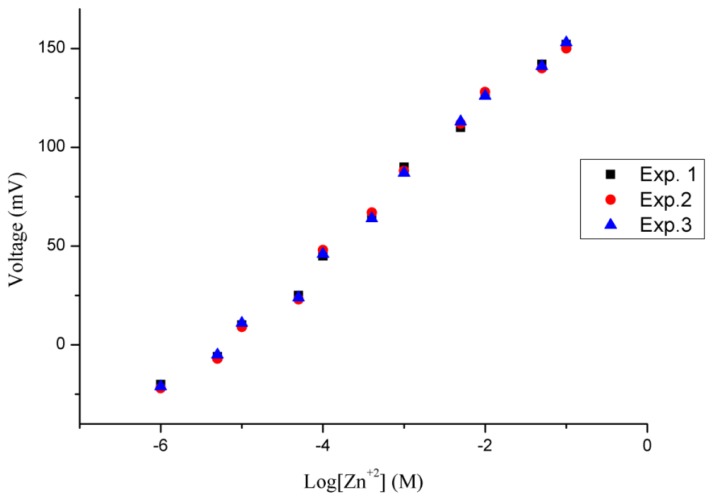
Repeatability of zinc ion sensor for 0.0005–100 mM zinc nitrate concentrations.

In this study, repeatability, reproducibility, and selectivity of the proposed ion selective electrode were examined. The repeatability of the ion selective electrode describes the response of a specific electrode which has been more than once under the same set of conditions. The functionalised NiO-based zinc ion selective electrode was tested for three consecutive days and it showed good repeatability with similar ranges of zinc ion detection, sensitivity and regression coefficient values as shown in Figure 5.

**Figure 6.**
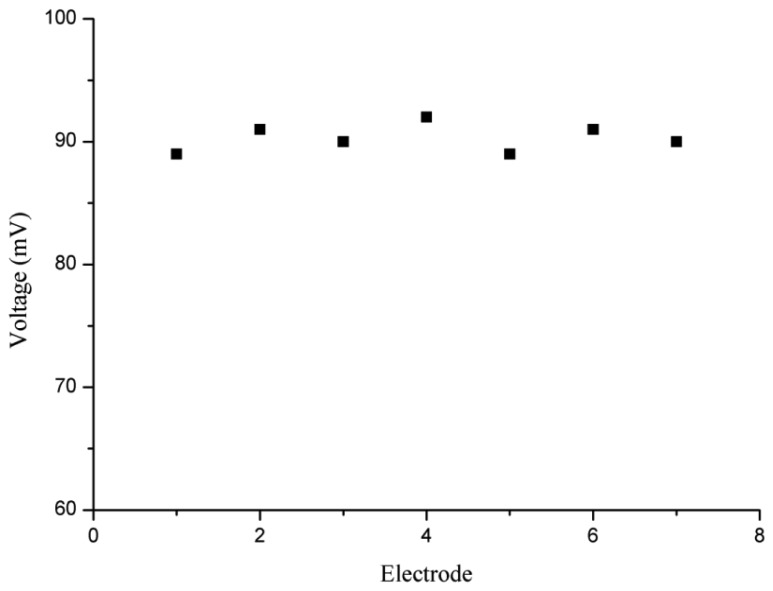
Reproducibility of zinc ion sensor in 0.1 mM solution of zinc nitrate.

For reproducibility, seven independent ion selective electrodes based on the honey- comb-like nanostructures of NiO were functionalised under the same conditions. All these zinc ion selective electrodes were used in a 0.1 mM solution of zinc nitrate electrolyte. It was observed that the proposed zinc ion selective sensor electrodes demonstrated high reproducibility with less than 5% standard deviation, as shown in Figure 6.

Selectivity of an ion selective sensor electrode is the fundamental parameter among all other parameters for the performance evaluation of an ion selective electrode. The separation solution method, which is recommended by IUPAC [52], was used for the study of the selectivity of the ion selective electrode by determination of the selectivity of coefficient values of both mono and divalent metal cations using 1 mM solution of each interferent. The calculated selectivity coefficient values are given in Table 1. The determined selectivity coefficient values are fairly constant and this study revealed that the proposed ion selective electrode is highly selective towards zinc ion.

### 3.4. Influence of Temperature on the Potentiometric Response of the NiO Nanostructures-Based Zinc Ion Sensor Electrode

The response of an ion selective electrode is also temperature dependent due to changes in the ionic mobility of target ions in solution. In this experiment, the effect of temperature on the response of the proposed electrode was observed from room temperature to 75 °C.

**Figure 7.**
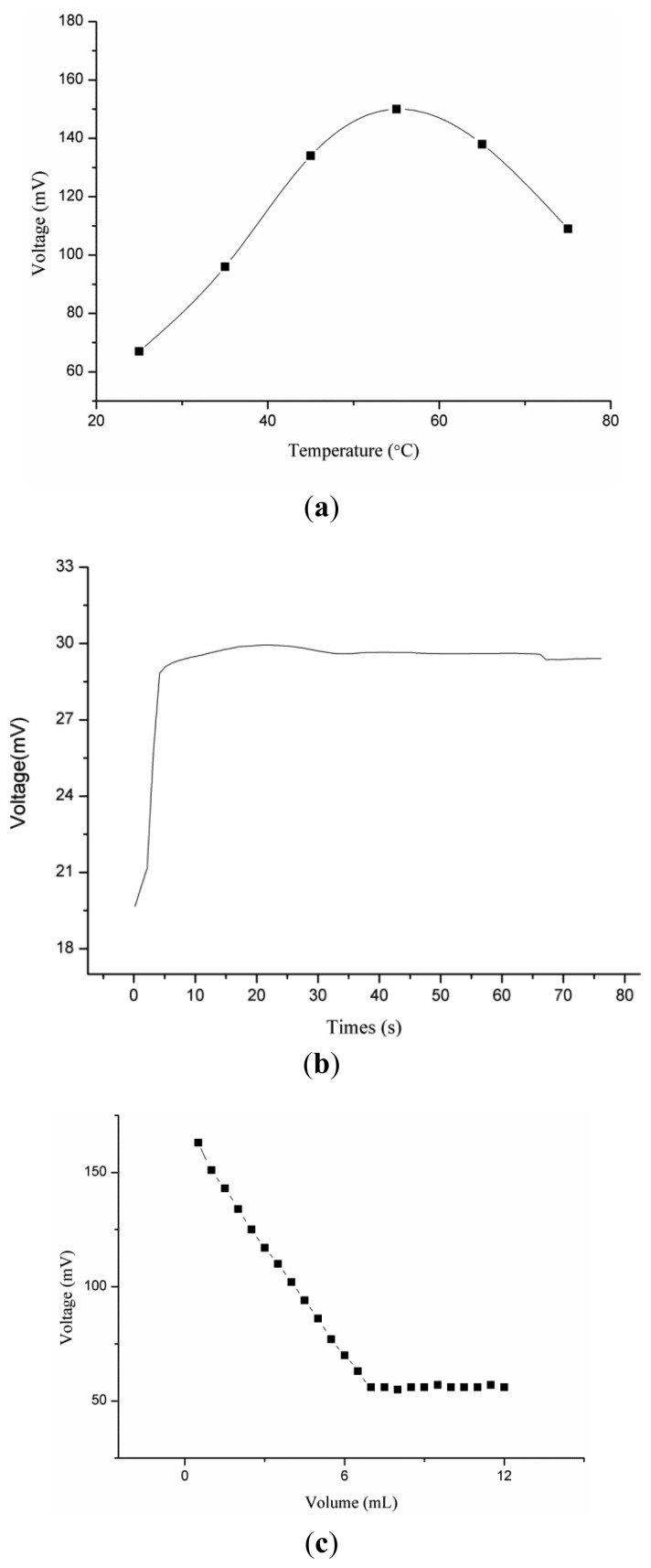
(**a**) Influence of temperature of the output response of zinc ion sensor. (**b**) Study of response time. (**c**) Potentiometric titration curve in 10 mM zinc nitrate solution.

It can be inferred from the Figure 7(a), that the output response was increasing gradually up to 55 °C due to the increase in the mobility of zinc(II) ion, but above 55 °C the response trend was increasing due to possible detachment of ionophore membrane from the surface of the honeycomb-like nanostructures of NiO and also at higher temperature the analyte ions suffered from self-resistance in the solution. Moreover, the proposed zinc ion sensor has shown response time of less than 10 s, as shown in Figure 7(b).

### 3.5. Analytical Application of the NiO Nanostructures-Based Zinc(II) Ion Selective Electrode

The functionalized NiO nanostructures-based ion selective electrode was used as indicator electrode for the potentiometric titration of 70 mL of 10 mM zinc(II) ions against 100 mM ethylenediaminetetraacetate solution [21] as shown in Figure 7(c). It can be seen from Figure 7(c) that with addition of EDTA the response of ion selective electrode was decreasing due to complex formation among zinc (II) ions and EDTA molecules, but at 7 mL a sharp intersection point was observed, which shows a good stoichiometric relationship for the determination of zinc ion concentration in unknown samples. Table 2 shows a comparison of the proposed zinc ion sensor based on the functionalised NiO honeycomb-like nanostructures with reported zinc ion sensors. The performance of the present zinc ion sensor is better than that of the reported zinc ion sensors due to the three dimensional network of NiO nanostructures and enhanced electrochemical properties of NiO in nanodimensional form.

## 4. Conclusions

In this work, honeycomb-like nanostructures of NiO were grown on nickel foam and also functionalised with 12-crown-4 a selective zinc ion ionophore. The as-grown NiO nanostructures exhibited good crystal quality and have shown a good potentiometric response in the development of a zinc ion-selective electrode. The NiO nanostructures-based zinc ion sensor electrode detected a wide linear range of zinc ion concentrations from 0.001 mM to 100 mM with a low limit of detection of 0.0005 mM. The sensitivity of the proposed zinc ion sensor was found to be of 36 mV/decade and the regression coefficient 0.99. Beside these characteristics, the zinc ion sensor electrode demonstrated good reproducibility, repeatability, and selectivity, a fast response time of less than 10 s and good storage stability. The zinc ion sensor electrode was also used as indicator electrode in potentiometric titrations. All the obtained results indicated that the proposed zinc ion sensor electrode has good potential for analysing zinc ions in industrial, clinical and other real samples.

## Figures and Tables

**Table 1.** Calculated selectivity values for different interferents.

**Interferent (X^+Z^)**	**Slope (mV/decade)**	$\log K_{Zn^{+2},X^{+z}}^{\text{pot}}$

K^+1^	2.40	−4.34
Co^+2^	4.30	−3.70
Mg^+2^	5.2	−4.71
Fe^+3^	7.6	−3.05
Na^+1^	7.5	−4.70
Ni^+2^	8.1	−4.72
Cu^+2^	3.6	−2.65

**Table 2.** The comprative study of present zinc ion sensor with the reported zinc ion sensors.

**S. No**	**Concentration Range**	**Slope sensitivity (mV/decade)**	**Response time (Seconds)**	**Reference**

**1**	0.006–100 mM	29.0	12	[24]
**2**	0.01–100 mM	35.0	5	[32]
**3**	0.013–100 mM	30.0	10	[53]
**4**	0.5–100 mM	33.0	20–25	[54]
**5**	0.005–100 mM	29.7	8	[55]
**6**	0.001–100 mM	36.0	Less than 10	this work
